# Effects of inositol in women with polycystic ovary syndrome: an umbrella review of meta-analyses from randomized controlled trials

**DOI:** 10.3389/fendo.2026.1741509

**Published:** 2026-02-11

**Authors:** Mengxue Duan, Min Yang, Chang Li, Xiao Wu, Xiaoxiao Yin, Hongqiu Zhu

**Affiliations:** 1 Chengdu University of Traditional Chinese Medicine, Chengdu, Sichuan, China; 2 Department of Gynecology, Hospital of Chengdu University of Traditional Chinese Medicine, Chengdu, Sichuan, China

**Keywords:** D-chiro-inositol, inositol, insulin resistance, myo-inositol, pcos, reproduction, umbrella review

## Abstract

**Objectives:**

This umbrella review aimed to synthesize and appraise the evidence regarding the efficacy of inositol for Polycystic Ovary Syndrome (PCOS) by integrating meta-analyses of randomized controlled trials(RCTs), thereby assessing the robustness of the existing body of evidence.

**Methods:**

We searched four databases from inception to August 2025 for relevant RCT meta-analyses. Primary outcomes included hormonal profiles, glycolipid metabolism, anthropometrics, and reproductive outcomes. Quality was assessed using AMSTAR-2 and GRADE.

**Results:**

Thirteen meta-analyses were included. AMSTAR-2 ratings were 23.1% high, 53.8% low, and 23.1% very low quality. GRADE assessment of 85 evidence items revealed no high-quality evidence; 18.9% were moderate, 40% low, and 41.1% very low quality. Pooled analyses demonstrated that inositol significantly improved multiple outcomes compared to placebo/FA: it reduced serum luteinizing hormone (LH: MD -3.43 IU/L, 95% CI [-4.29, -2.56], *P* < 0.00001), total testosterone (TT), free testosterone (FT: MD -0.02 nmol/L, 95% CI [-0.02, -0.01], *P* < 0.00001), improved sex hormone-binding globulin (SHBG: MD 36.72 nmol/L, 95% CI [28.52, 44.91], *P* < 0.00001), and androstenedione. Benefits were also observed for homeostatic model assessment of insulin resistance (HOMA-IR: MD -1.14, 95% CI [-1.35, -0.94], *P* < 0.00001), fasting insulin (FI: MD -23.40 pmol/L, 95% CI [-32.80, -14.01], *P* < 0.00001), triglycerides, and reproductive outcomes (live births: Risk Ratio [RR] 2.29, 95% CI [1.07, 4.93], *P* = 0.03; ovulation rate: RR 2.75, 95% CI [1.71, 4.41], *P* < 0.0001). However, versus metformin(MET), its effects on most parameters were not significant, except for triglycerides and pregnancy rates. Cross-subgroup analysis of inositol subtypes indicated MI/MI+FA was superior for metabolic and reproductive outcomes, while D-chiro-inositol monotherapy should be used with caution in clinical practice; combination therapy did not consistently outperform monomers.

**Conclusion:**

Inositol improves core PCOS manifestations. Supported by moderate-quality evidence for effects on TT, FT, SHBG, HOMA-IR, and pregnancy/ovulation rates, it is a promising therapy. Differential efficacy of inositol subtypes may inform personalized treatment. However, outcomes based on low-quality evidence require cautious interpretation and should not solely guide clinical decisions, highlighting the need for larger, rigorous trials.

**Systematic Review Registration:**

https://www.crd.york.ac.uk/prospero/, identifier CRD420251146691.

## 1 Introduction

Polycystic ovary syndrome (PCOS) is a classic endocrine-metabolic disorder characterized by persistent anovulation, hyperandrogenism, and polycystic ovarian changes. Common clinical complications closely associated with this condition include metabolic disorders such as obesity and insulin resistance (IR) (1). Over the past three decades, the incidence and prevalence of polycystic ovary syndrome have shown a significant upward trend, with approximately 10% to 13% of the global population currently affected by this condition (2). The pathogenesis of PCOS is multifactorial, resulting from the complex interplay among genetic susceptibility, environmental factors, and transgenerational effects. This etiological complexity underlies a broad spectrum of clinical manifestations that interlink metabolic disturbances, reproductive dysfunction, and psychological challenges. A central reproductive concern in PCOS is infertility, which is predominantly caused by anovulation. Indeed, PCOS is responsible for approximately 70% of all anovulatory infertility cases (3).Furthermore, individuals with PCOS are at an increased lifelong risk of obesity, metabolic syndrome, type 2 diabetes (4), and coronary artery disease (5).

Treatment strategies for PCOS primarily involve lifestyle modifications and pharmacological interventions. For mild symptoms, lifestyle changes are recommended. Pharmacological treatment is determined by symptom severity, targeting IR, oligoovulation, and hyperandrogenism. Primary medications include MET, clomiphene citrate, and oral contraceptives (6).Due to the complexity of PCOS, developing personalized treatment plans presents a significant challenge in clinical practice. Research indicates that nutritional supplements demonstrate remarkable clinical efficacy in treating PCOS (7). Studies have demonstrated that consuming inositol helps lower insulin resistance and enhance metabolic parameters among individuals diagnosed with PCOS. Its therapeutic effects are derived from its participation in multiple biological processes, including insulin and gonadotropin signal transmission, energy metabolism, follicular growth, glucose balance, cell movement, and neural tube development. Additionally, inositol plays a critical role in supporting fertility and ensuring normal embryonic development (8, 9).

Although several meta-analyses have examined the effects of inositol interventions on PCOS, there remains a lack of systematic, integrated research to comprehensively assess the strength, heterogeneity, and methodological quality of this evidence across key clinical domains such as hormone levels, metabolic markers, anthropometric parameters, and reproductive outcomes. Therefore, this umbrella review aims to systematically synthesize existing meta-analysis evidence, assess its methodological quality using the AMSTAR-2, and grade the certainty of evidence through the GRADE. This approach aims to establish a robust evidence to fill current knowledge gaps and provide a basis for clinical decision-making and future research directions.

## 2 Materials and methods

### 2.1 Search strategy and study selection

The methodological framework of this umbrella review adhered to the PRISMA-P guidelines (10) and has been registered in PROSPERO (CRD420251146691). We systematically searched PubMed, Scopus, Embase, and Web of Science from inception through August 2025 using predefined terms related to Polycystic Ovary Syndrome (“Polycystic Ovary Syndrome”, “Stein-Leventhal Syndrome”, “Inositol”, “Chiro-Inositol”, “MyoInositol”, “Meta-Analysis”, “meta-analyses”, “meta-analyze” and “Systematic Review”).Boolean operators (OR for synonyms, AND for paratactic terms were employed without language restrictions(**Supplementary Table S2**).

Two researchers (M.X.D.and M.Y.) screened the titles and abstracts of studies individually, then systematically evaluated full texts and manually verified references according to inclusion criteria.Disagreements got resolved via consultation with a third researcher (X.W.).

### 2.2 Eligibility criteria

The meta-analyses included in this umbrella review, which focused on inositol, were all based on randomized controlled trials. The inclusion process for all studies strictly adhered to the PICOS principles*(***Table 1**). Exclusion criteria were as follows (1): studies where subjects received other interventions; (2) *in vivo* and *in vitro* studies, case reports, observational studies, and quasi-experimental designs, (3) review studies with no quantitative analysis, and (4) systematic reviews lacking meta-analyses and (5) those failing to provide comprehensive data.

**Table 1:** PICOS criteria for inclusion of studies.

Parameter	Criterion
Population	Adult women with PCOS
Interventions	Inositol(MI, DCI, MI+DCI combination)
Comparators	Placebo, no treatment or any active intervention (e.g. an insulin-sensitising agent, an ovulation induction agent)
Outcomes	Hormonal and glycolipid profiles, anthropometric data, reproductive outcomes

### 2.3 Data extraction

The two researchers (M.X.D. and M.Y.) independently completed the data extraction, with the final dataset confirmed by having all discrepancies examined and adjudicated through consensus by a third researcher(C.L.) consulting the original materials. Data items collected comprised first author, publication year, country/region, number of included studies, sample size, details of intervention and control groups, study duration, outcome measures, weighted mean difference (MD), risk ratio (RR), and their corresponding 95% confidence intervals (CI).

### 2.4 Methodological and evidence quality

AMSTAR-2, a valid instrument for umbrella reviewst (11), was used to assess the methodological quality of each included meta-analysis.The evaluator answers with a “yes”, “partially yes” or “no” to each item, based on the content of the systematic review report.

By using the 16-item checklist and particularly paying attention to screening for “critical flaws” in these 7 items, one makes a qualitative judgment of confidence in the systematic review labeled as “High, “ “Moderate, “ “Low, “ or “Very Low.”The certainty of evidence for all outcomes of this umbrella review was graded using the GRADE framework (12). Finally, the evidence was designated as existing at one of the following four recommendation grades: high, moderate, low, or very low.

### 2.5 Data analyses

This umbrella review aims to synthesize existing meta-analysis evidence on inositol treatment for PCOS. All included meta-analyses exclusively comprised RCTs. This study aims to provide a descriptive synthesis and quality assessment of existing evidence rather than conduct a new meta-analysis. All pooled effect sizes were derived from a secondary synthesis of relevant meta-analysis results to systematically present the overall trend of evidence in this field. MD with 95% CI were applied in statistical analysis for continuous variables; RR with 95% CI were used in statistical analysis for categorical variables. All the meta-analyses used random-effects model. The statistical heterogeneity was evaluated by I² statistic and Cochran’s Q test, and the large heterogeneity was indicated when I² value was more than 50%. The significance level of heterogeneous test was *P* < 0.10. Because there were less than 10 included studies for each item of outcome measurement, the small-study effects analysis was not performed (13). The umbrella review was performed based on Review Manager 5.4 version (Cochrane Collaboration, Oxford, UK).

## 3 Results

### 3.1 Characteristics of the included studies

**Figure 1:**
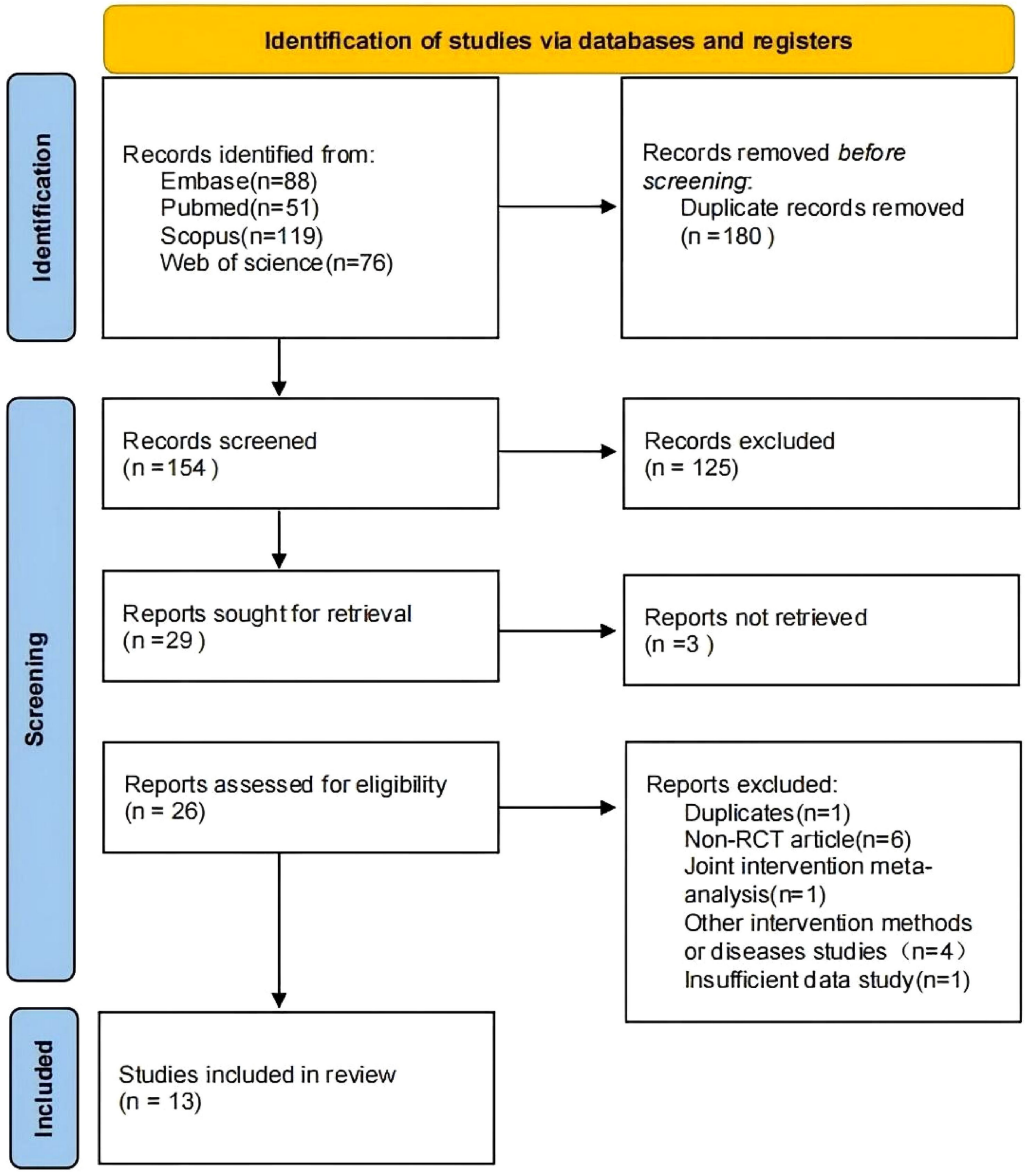
Flow diagram of the study search and selection process.

334 records from four databases were identified. After the exclusion of 180 duplicates, 125 studies were excluded by screening titles and abstracts, and 13 studies by full-text review.This study included meta-analyses published between 2017 and 2022. Sample sizes, measured in number of RCTs included in each meta-analysis, ranged from 2 to 35. Study selection flow of the included meta-analyses is shown in **Figure 1**. Characteristics of the included studies are presented in detail in **Table 2**. The specific definitions of the treatment group and control group are as follows **Table 3**, and subsequent results reporting will present and compare data grouped according to the above definitions,with all findings displayed in **Table 4**.

**Table 2:** Characteristics of included studies.

Citation (First author, year)	Country	No. of RCTs in Meta-analysis	Sample size	Intervention	Control	Duration	Quality assessment scale and rating
Arentz, 2017 (14)	Australia	8	327	Myo-Inositol(MI)/MI+FA or D-chiro-inositol(DCI)	Placebo/FA/MET	6–20 weeks	Yes (Cochrane)8/8 were low
Facchinetti, 2019(16)	Italy	6	355	MI/MI+FA	MET	12–24 weeks	Yes (Cochrane)6/6 were moderate
Fatima, 2023 (20)	Pakistan	8	1088	MI/MI+FA	MET	12–24 weeks	Yes (Cochrane)3/8 were high
Greff, 2023 (17)	Hungary	24	1691	1. MI/MI+FA 2. DCI 3. (MI/MI+FA)+DCI 4. Combination of the Three	Placebo/FA/Met/Diet	7–24 weeks	Yes (Cochrane)8/24 were high
Jethaliya, 2022 (25)	India	17	1083	MI/MI+FA	FA/MET/Oral contraceptive pill (OCP)	12–48 weeks	Yes (Cochrane) 5/17 were high
Mendoza, 2017 (21)	Spain	8	1019	MI/MI+FA	Ovulation-inducing agent/no	8–12 weeks	Yes (Cochrane) 2/8 were high
Morley, 2017 (24)	UK	2	371	DCI	Placebo	6–8 weeks	Yes (Cochrane) 1/2 were low
Pundir, 2017 (18)	UK	10	601	1. MI/MI+FA 2. (MI/MI+FA) or DCI	Placebo/no treatment/MET/DCI	8–12 weeks	Yes (Cochrane) 10/10 were moderate
Showell, 2018 (22)	New Zealand	13	1472	MI	Placebo, no treatment/standard, melatonin, MET, clomiphene citrate/DCI	8-24weeks	Yes (Cochrane) 4/13 were low
Unanyan, 2022 (23)	UK	35	4668	MI	Placebo/no treatment/FA/MET	4–12 weeks	Yes (Cochrane) 8/35 were low
Unfer, 2017 (26)	Switzerland	9	496	1. MI/MI+FA 2. (MI/MI+FA)+DCI	FA/DCI/OCP	12–24 weeks	Yes (Cochrane) 2/9 were low
Zeng, 2018 (15)	China	10	573	1.MI/MI+FA 2.(MI/MI+FA)+DCI	Placebo/FA/MET/OCP/DCI	12–24 weeks	Yes (Cochrane) 3/9 were low
Zhang, 2022 (19)	China	9	612	MI/MI+FA	MET	3–6 weeks	Yes (Cochrane) 2/9 were low

**Table 3:** Definition of inositol intervention and control groups.

Type	Definition
Intervention Group	
Iinositol	A combination of the following 1–4 conditions
MI	Intervention using myo-inositol alone.
DCI	Intervention using D-chiro-inositol alone.
MI+FA	Combined intervention of myo-inositol and folic acid.
MI+DCI	Combined intervention of myo-inositol and D-chiro-inositol.
Control Group	
Placebo/FA	Includes control groups receiving placebo alone, FA alone, or placebo combined with FA. These controls primarily assess the absolute efficacy of MI relative to inactive or baseline supplements.
MET	Control group receiving metformin alone. These comparisons aim to evaluate the relative efficacy of inositol compared to a standard insulin-sensitizing agent.
Other specific controls	Such as ovulation-inducing drugs, DCI (as a control), OCP, etc., will be clearly labeled in the results.

**Table 4:** The effects of inositol on all outcomes in PCOS women.

Outcomes stratified by different covariates	No. studies	Intervention	Control	Pooled effect size(WD/RR) (95% CI)	*P*-value	I^2^ (%)	*P*-heterogeneity	Evidence certainty (GRADE^1^)
Hormonal outcomes								
LH (IU/L)	2	Inositol	Placebo/FA	-3.43 [-4.29, -2.56]	<0.00001	0	0.89	Low
FSH (IU/L)	2	Inositol	Placebo/FA	-1.24 [-1.70, -0.78]	<0.00001	52	0.15	Very low
TT (nmol/L)	5			-0.30 [-0.59, -0.00]	0.05	72	0.002	Low
	3	Inositol	Placebo/FA	-0.91 [-1.29, -0.53]	<0.00001	0	0.92	Moderate
	3	MI/MI+FA	MET	0.02 [-0.10, 0.15]	0.71	0	0.94	Low
	1	DCI	Place/FA	-1.45 [-2.43, -0.47]	0.004	/	/	Low
FT (nmol/L)	4			-0.02 [-0.02, -0.01]	<0.00001	32	0.22	Moderate
		Inositol	Place/FA	-0.02 [-0.02, -0.02]	0.0003	78	0.0004	Low
		DCI	Place/FA	-0.02 [-0.03, -0.01]	<0.00001	0	0.88	Moderate
		(MI/MI+FA)+DCI	Place/FA	-0.00 [-0.01, 0.00]	0.0002	/	/	Low
Androstenidione (nmol/L)	5			-1.84 [-3.25, -0.44]	0.01	85	<0.00001	Low
	4	Inositol	Place/FA	-2.67 [-3.68, -1.66]	<0.00001	43	0.15	Moderate
	1	MI/MI+FA	MET	-0.05 [-0.65, 0.55]	0.87	/	/	Low
	1	DCI	Place/FA	-1.81 [-3.63, -0.00]	0.05	/	/	Low
	1	(MI/MI+FA)+DCI	Place/FA	0.42 [-4.54, 5.38]	0.87	/	/	Very low
SHBG (nmol/L)	6			17.57 [5.53, 29.61]	0.004	91	<0.00001	Low
	4	Inositol	Place/FA	36.72 [28.52, 44.91]	<0.00001	0	0.59	Moderate
	3	MI/MI+FA	MET	-2.81 [-11.28, 5.65]	0.51	79	0.008	Very low
	1	DCI	Place/FA	55.45 [25.99, 84.91]	0.0002	/	/	Low
	1	(MI/MI+FA)+DCI	Place/FA	10.82 [-1.70, 23.34]	0.09	/	/	Very low
DHEAS (µmol/L)	3			-1.84 [-4.25, 0.57]	0.13	92	<0.00001	Very low
	3	Inositol	Place/FA	-3.58 [-3.99, -3.17]	<0.00001	0	0.64	Moderate
	1	MI/MI+FA	MET	0.47 [-0.49, 1.43]	0.34	/	/	Low
	1	DCI	Place/FA	-4.57 [-7.63, -1.51]	0.003	/	/	Low
	1	(MI/MI+FA)+DCI	Place/FA	1.15 [-2.43, 4.74]	0.53	/	/	Very low
Glycaemic factors								
FG (mmol/l)	5			-0.34[-3.46, 2.78]	0.83	86	<0.00001	Very low
	3	Inositol	Placebo/FA	-0.78[-5.68, 4.12]	0.76	93	<0.00001	Very low
	2	MI/MI+FA	MET	0.13[-2.52, 2.78]	0.92	0	0.36	Low
FI (pmol/L)	6			-10.92[-17.69, -4.16]	0.002	93	<0.00001	Low
	3	Inositol	Placebo/FA	-23.40[-32.80, -14.01]	<0.00001	68	0.04	Low
	3	MI/MI+FA	MET	-0.45[-1.73, 0.83]	0.49	0	0.78	Low
HOMA-IR	7			-0.52 [-0.89, -0.16]	0.005	92	<0.00001	Low
	4	Inositol	Place/FA	-1.14 [-1.35, -0.94]	<0.00001	11	0.34	Moderate
	4	MI/MI+FA	MET	-0.12 [-0.31, 0.08]	0.24	66	0.03	Low
	1	(MI/MI+FA)+DCI	Place/FA	-0.73 [-2.20, 0.74]	0.33	/	/	Very low
AUC Glucose (mmol.min/L)	2			-54.98 [-139.28, 29.33]	0.2	94	<0.00001	Very low
	2	Inositol	Place/FA	-85.84 [-192.69, 21.00]	0.12	97	<0.00001	Very low
	1	MI/MI+FA	MET	67.64 [-45.12, 180.40]	0.24	/	/	Very low
	1	DCI	Place/FA	-83.38 [-189.04, 22.28]	0.12	/	/	Very low
AUC insulin (pmol.min/L)	2			-16212.32 [-23800.77, -8623.87]	<0.0001	66	0.03	Very low
	2	Inositol	Place/FA	-17571.05 [-24229.85, -10912.25]	<0.00001	77	0.04	Very low
	1	MI/MI+FA	MET	9562.26 [-16811.94, 35936.46]	0.48	/	/	Very low
	1	DCI	Place/FA	-27968.70 [-58008.85, 2071.45]	0.07	/	/	Very low
Lipid profiles								
Cholesterol (mmol/l)	2			0.25[-0.22, 0.71]	0.3	71	0.07	Very low
	1	Inositol	Placebo/FA	0.56[0.04, 1.08]	0.03	/	/	Low
	1	MI/MI+FA	MET	0.07[-0.04, 0.17]	0.23	/	/	Very low
Triglycerides (mmol/l)	2			-0.16[-0.44, 0.12]	0.27	68	0.08	Very low
	1	Inositol	Placebo/FA	-0.36[-0.69, -0.02]	0.04	/	/	Low
	1	MI/MI+FA	MET	-0.06[-0.09, -0.02]	0.001	/	/	Low
HDL(mmol/l)	2			-0.02[-0.12, 0.08]	0.69	0	0.56	Very low
	1	Inositol	Placebo/FA	0.00[-0.12, 0.12]	1	/	/	Very low
	1	MI/MI+FA	MET	-0.06[-0.24, 0.11]	0.48	/	/	Very low
LDL(mmol/l)	2			0.05[-0.08, 0.19]	0.44	0	0.44	Very low
	1	Inositol	Placebo/FA	-0.05[-0.35, 0.25]	0.74	/	/	Very low
	1	MI/MI+FA	MET	0.08[-0.07, 0.23]	0.3	/	/	Very low
Anthropometric outcomes								
BMI(kg/m²)	5			0.02 [-0.31, 0.35]	0.92	32	0.17	Low
	2	Inositol	Placebo/FA	-0.44[-0.82, -0.06]	0.02	0	0.91	Moderate
	4	Inositol	MET	0.29[-0.27, 0.86]	0.31	52	0.1	Very low
	1	DCI	MET	0.35 [-0.57, 1.27]	0.45	/	/	Very low
	1	(MI/MI+FA)+DCI	Place/FA	-0.21 [-4.44, 4.02]	0.92	/	/	Very low
WHR	2			-0.00 [-0.04, 0.03]	0.8	72	0.06	Very low
	1	Inositol	Placebo/FA	-0.02 [-0.03, -0.01]	<0.0001	/	/	Low
	1	Inositol	MET	0.02 [-0.02, 0.06]	0.33	/	/	Low
Reproduction outcomes								
Pregnancy rates/Clinical pregnancy	6			1.29 [1.11, 1.50]	0.0008	33	0.12	Moderate
	5	INositol	placebo/FA	1.30 [0.95, 1.76]	0.1	29	0.23	Moderate
Outcomes stratified by different covariates	No.studies	Intervention	Control	Pooled effect size(WD/RR)(95% CI)	*P*-value	I^2^(%)	*P*-heterogeneity	Evidencecertainty(GRADE^1^)
	4	INositol	MET	1.43 [1.14, 1.79]	0.002	0	0.77	Moderate
	1	INositol	Ovulation-inducing agent/no	1.19 [0.91, 1.55]	0.2	/	/	Low
	1	(MI/MI+FA)+FA	antioxidant(melatonin)	0.88 [0.66, 1.18]	0.39	/	/	Moderate
	1	MI/MI+FA	DCI	2.86 [1.14, 7.17]	0.03	/	/	Moderate
	1	(MI/MI+FA)+DCI	Place/FA	1.45 [1.06, 1.98]	0.02	/	/	Low
Live births	2			1.58[0.95, 2.63]	0.08	0	0.42	Low
	2	Inositol	Placebo/FA	2.29[1.07, 4.93]	0.03	0	0.75	Low
	1	MI/MI+FA	Ovulation induction agent(clomiphene)	1.18[0.60, 2.33]	0.63	/	/	Moderate
Ovulation rate	4			1.36 [1.04, 1.77]	0.03	73	0.002	Low
	2	Inositol	Placebo/FA	2.75[1.71, 4.41]	<0.0001	0	0.53	Moderate
	2	MI/MI+FA	MET	1.33[0.99, 1.77]	0.05	0	0.73	Moderate
	1	MI/MI+FA	Ovulation induction agent(clomiphene)	0.87[0.67, 1.13]	0.3	/	/	Moderate
Combination research								
TT (nmol/L)	3	Inositol	Placebo/FA/MET/OCP	-0.40[-0.68, -0.12]	0.005	0	0.88	Low
SHBG(nmol/L)	2	Inositol	Placebo/FA/MET/OCP	5.40[-14.96, 25.76]	0.6	78	0.03	Very low
DHEAS(µmol/L)	2	Inositol	Placebo/FA/MET/OCP	-0.92[-2.05, 0.22]	0.11	0	0.83	Very low
FI(pmol/L)	3	Inositol	Placebo/FA/MET/OCP	-15.39[-22.80, -7.98]	<0.0001	30	0.24	Low
FG(mmol/l)	2	Inositol	Placebo/FA/MET/OCP	0.03[-0.10, 0.16]	0.64	0	0.4	Very low
HOMA-IR	3	Inositol	Placebo/FA/MET/OCP	-0.54[-0.79, -0.29]	<0.0001	0	0.67	Low
BMI(kg/m²)	2	Inositol	Placebo/FA/MET/OCP	-0.04[-0.73, 0.66]	0.92	39	0.2	Very low

^1GRADE certainty of evidence: High = further research is very unlikely to change our confidence in the effect estimate; Moderate = further research is likely to have an important impact; Low = further research is very likely to have an important impact; Very low = any estimate is very uncertain. Downgrading reasons included risk of bias,heterogeneity,indirectness,imprecision,and suspected publication bias.^

### 3.2 Effects of inositol on hormonal parameters

Seven sex hormone-related outcomes were analyzed from six published studies.

Two studies (14, 15) demonstrated that inositol significantly reduced LH (MD: -3.43, 95% CI: [-4.29, -2.56], *P* < 0.00001 I^2^ = 0%, *P*-heterogeneity = 0.89, GRADE: Low) and FSH (MD: -1.24, 95% CI: [-1.70, -0.78], *P* < 0.00001, I^2^ = 52%, *P*-heterogeneity = 0.15, GRADE: Very low).

Analysis of TT from five studies (15–19) showed a small overall reduction (MD: -0.20, 95% CI: [-0.46, 0.06], *P* = 0.14, I^2^ = 61%, *P*-heterogeneity = 0.02, GRADE: Low), which lacked statistical significance; notably, this result was confounded by substantial heterogeneity constraining its interpretability. The effect was more pronounced when compared with placebo/folic acid(FA) (MD: -0.62, 95% CI: [-1.12, -0.11], *P* = 0.02, I^2^ = 0%, *P*-heterogeneity = 0.5, GRADE: Moderate), but no significant difference compared to MET. For DCI vs. placebo/FA, TT was markedly reduced (MD -1.45 95% CI: [-2.43, -0.47], *P* = 0.004, GRADE: Low). Four studies (14, 15, 17, 18) indicated that inositol significantly reduced FT (MD: -0.02, 95% CI: [-0.02, -0.01], *P* < 0.00001, I^2^ = 32%, *P*-heterogeneity = 0.22, GRADE: Moderate). Similarly, DCI vs. placebo/FA yielded a significant FT reduction (MD: -0.02 95% CI:[-0.03, -0.01], *P* = 0.0002, GRADE: Low). The efficacy of MI + DCI combination therapy was not statistically significant.

Five studies (14–18) showed that inositol significantly reduced androstenedione (pooled MD: -1.84, 95% CI: [-3.25, -0.44], *P* = 0.01, I^2^ = 85%, *P*-heterogeneity < 0.00001, GRADE: Low), but this result was characterized by very high heterogeneity and thus requires cautious interpretation. A clearer effect was seen in the placebo/FA subgroup (MD: -2.67, 95% CI: [-3.68, -1.66], *P* < 0.00001, I^2^ = 43%, *P*-heterogeneity = 0.15, GRADE: Moderate), with no significant difference compared with MET. In the DCI vs. placebo/FA subgroup, androstenedione concentrations exhibited a marked decrease (MD: -1.81, 95% CI: [-3.63, -0.00], *P* = 0.05, GRADE: Low).The efficacy of MI + DCI combination therapy was not statistically significant.

Six studies (14–19) indicated that inositol significantly increased SHBG (overall MD: 17.57, 95% CI: [5.53, 29.61], *P* = 0.004, I^2^ = 91%, *P*-heterogeneity < 0.00001, GRADE: Low), though this finding was also marked by considerable heterogeneity. Subgroup analysis revealed that the placebo/FA group demonstrated more pronounced efficacy with lower heterogeneity (MD: 36.72, 95% CI: [28.52, 44.91], *P* < 0.00001, I^2^ = 0%, *P*-heterogeneity = 0.59, GRADE: Moderate), with no significant difference compared to MET. In contrast, the DCI vs. placebo/FA subgroup showed a significant decrease in SHBG (MD: 55.45, 95% CI:[25.99, 84.91], P = 0.0002, GRADE: Low). No statistically significant effect was observed with MI + DCI combination therapy. Finally, three studies (15, 17, 18) showed that inositol significantly reduced dehydroepiandrosterone sulfate (DHEAS) compared with placebo/FA (MD: -3.58, 95%CI: [-3.99, -3.17], *P* < 0.00001, I^2^ = 0%, *P*-heterogeneity = 0.64, GRADE: Moderate), with no significant difference overall or compared with MET. The DCI vs. placebo/FA subgroup also showed a significant decrease in DHEAS (MD: -4.57, 95% CI: [-7.63, -1.51], *P* = 0.003, GRADE: Low), while MI + DCI combination therapy did not yield statistically significant results.

### 3.3 Effects of inositol on glycemic parameters

Five glycemic parameters were extracted from the included studies: Fasting Glucose (FG), Fasting Insulin (FI), HOMA-IR, area under curve(AUC) Glucose, and AUC Insulin. Detailed data are presented in the **Table 4**.

For FI, six studies (14–16, 18–20) demonstrated that inositol significantly reduced FI overall (MD: -10.92, 95% CI: [-17.69, -4.16], *P* = 0.002, I^2^ = 93%, *P*-heterogeneity < 0.00001, GRADE: Low), but with substantial heterogeneity. A stronger reduction was observed vs. placebo/FA(MD: -23.40, 95% CI: [-32.80, -14.01], *P* < 0.00001, I^2^ = 68%, *P*-heterogeneity = 0.04, GRADE: Low) and no significant difference in the MET subgroup.

Seven studies (14–20) showed that inositol significantly improved HOMA-IR overall (MD: -0.51, 95% CI: [-0.88, -0.14], *P* = 0.007, I^2^ = 93%, *P*-heterogeneity < 0.00001, GRADE: Low), though this was accompanied by high heterogeneity. The subgroup analysis against Placebo/FA showed a significant effect with no substantial heterogeneity (MD: -1.09, 95% CI: [-1.35, -0.83], *P* < 0.00001, I^2^ = 40%, *P*-heterogeneity = 0.17, GRADE: Moderate). No significant effect was observed in the MET subgroup.

Regarding AUC Insulin, two studies (17, 18) indicated that inositol overall significantly reduced AUC Insulin (MD:-16212.32, 95% CI: [-23800.77, -8623.87], *P* < 0.0001, I^2^ = 66%, *P*-heterogeneity = 0.03, GRADE: Very low), with significant reduction in the Placebo/FA subgroup (MD: -17571.05, 95% CI: [-24229.85, -10912.25], *P* < 0.00001, I^2^ = 77%, *P*-heterogeneity = 0.04, GRADE: Very low), though both results are supported by very low-quality evidence and considerable heterogeneity. But no significant difference in the MI vs. MET and DCI vs. placebo/FA subgroup.

FG (14, 15, 18–20) and AUC Glucose (17, 18) levels did not show significant improvement with inositol treatment.

### 3.4 Effects of inositol on lipid profiles

Only two studies (14, 19) reporting lipid parameters (Cholesterol, Triglycerides, HDL, LDL) were included. As one study (14) used Placebo/FA and the other (19) used MET as the control, subgroup analyses were performed for each parameter.

Inositol therapy did not significantly increase high-density lipoprotein (HDL) or low-density lipoprotein (LDL) levels, regardless of the intervention compared. For Cholesterol, a significant effect was found specifically in the subgroup comparing inositol to Placebo/FA (MD: 0.56, 95% CI: [0.04, 1.08], *P* = 0.03, GRADE: Low). Neither the overall pooled estimate nor the comparison with MET showed a statistically significant effect.

Analysis of Triglycerides revealed a non-significant overall pooled effect. However, significant reductions were observed in both subgroup analyses (Inositol vs. Placebo/FA: MD: -0.36, 95% CI: [-0.69, -0.02], *P* = 0.04, GRADE: Low; MI/MI+FA vs. MET: MD: -0.06, 95% CI: [-0.09, -0.02], *P* = 0.001, GRADE: Low).

### 3.5 Effects of inositol on anthropometric outcomes

The evaluated anthropometric parameters comprised Body Mass Index (BMI) and Waist-to-Hip Ratio (WHR). Five (15–17, 19, 20) studies reported BMI, and two studies (14, 19) reported WHR. Only the result in the WHR subgroup(inositol to Placebo/FA) was statistically significant (WHR: MD: -0.02, 95% CI: [-0.03, -0.01], *P* < 0.0001, GRADE: Low).

### 3.6 Effects of inositol on reproductive outcomes

Reproductive outcomes included Pregnancy rates/Clinical pregnancy, Live births, and Ovulation rate.

For Pregnancy rates/Clinical pregnancy, six studies (14, 17, 18, 21–23) were included and analyzed across six subgroups. Inositol significantly increased pregnancy rates overall and in subgroups compared with Placebo/FA, MET and DCI. Moreover, MI+DCI demonstrated a statistically significant superiority over placebo. No significant differences were observed in the remaining subgroups.Specific data: Overall: RR: 1.29, 95% CI: [1.11, 1.50], *P* = 0.001, I^2^ = 30%, *P*-heterogeneity = 0.15; Inositol vs. Placebo/FA: RR: 1.31, 95% CI: [1.05, 1.63], *P* = 0.02, I^2^ = 9%, *P*-heterogeneity = 0.35; Inositol vs. MET: RR: 1.43, 95% CI: [1.14, 1.79], *P* = 0.002, I^2^ = 0%, *P*-heterogeneity = 0.77; MI vs. DCI: RR:2.86, 95% CI: [1.14, 7.17], *P* = 0.03; MI+DCI vs. Place/FA: RR:1.45, 95% CI:[1.06, 1.98], *P* = 0.02. Except for the last group, which had low GRADE evidence, the quality of evidence for all other control groups was moderate.

For Live births, two studies (14, 22) were included, (one study contained both Placebo/FA and MET control groups). Only the Inositol vs. Placebo/FA subgroup showed a significant increase in live birth rate (RR: 2.29, 95% CI: [1.07, 4.93], *P* = 0.03, I^2^ = 0%, *P*-heterogeneity = 0.75, GRADE: Low), though this is based on limited studies and low-quality evidence. And no significant differences were observed overall or in the vs. MET subgroup.

Four studies reported on Ovulation rate (14, 18, 22, 24), divided into four subgroups. Inositol significantly increased ovulation rate overall and in the following subgroups: Inositolvs Placebo/FA, MI/MI+FA vs. MET, DCI vs placebo/no treatment. No significant differences were observed in the remaining subgroups. Specific data:(Overall: RR: 1.36, 95% CI: [1.04, 1.77], *P* = 0.03, I^2^ = 73, *P*-heterogeneity =0.002, GRADE: Low; Inositol vs. Placebo/FA: RR: 2.75, 95% CI: [1.71, 4.41], *P* < 0.0001, I^2^ = 0%, *P*-heterogeneity = 0.53, GRADE: Moderate; MI/MI+FA vs. MET: RR: 1.33, 95% CI: [0.99, 1.77], *P* = 0.05, I^2^ = 0%, *P*-heterogeneity = 0.73, GRADE: Moderate; DCI VS. Placebo/No treatment: RR: 1.16, 95% CI: [1.07, 1.26], *P* = 0.0004, GRADE: Low). The other subgroups did not show statistically significant differences.

Additionally, three meta-analyses (15, 25, 26) using the same control groups were incorporated. Two studies (25, 26) combined control group data in their reports without providing subgroup analyses stratified by intervention; another study also reported pooled comparisons for the same control groups. Given the consistent data reporting approach across these three studies, we combined their reported results(TT, DHEAS, SHBG, FI, FG, HOMA-IR, and BMI) for analysis. Three meta-analyses contributed to TT, FI, and HOMA-IR, while two contributed to the other outcomes. Pooled analysis of these meta-analyses indicated significant improvements for TT, FI, and HOMA-IR with inositol treatment, with no heterogeneity (TT: MD: -0.40, 95% CI: [-0.68, -0.12], *P* = 0.005, I^2^ = 0%, *P*-heterogeneity = 0.88; FI: MD: -15.39, 95% CI: [-22.80, -7.98], *P* < 0.0001, I^2^ = 30%, *P*-heterogeneity = 0.24; HOMA-IR: MD: -0.54, 95% CI: [-0.79, -0.29], *P* < 0.0001, I^2^ = 0%, *P*-heterogeneity = 0.67). The evidence for these three outcomes was rated as low certainty. The remaining outcomes showed no statistically significant effects.

### 3.7 Heterogeneity and sensitivity analysis

The I^2^ statistic and Cochran’s Q test were reported for all meta-analyses (n = 13) and all estimated summary effects (n = 85), as detailed in the **Table 4**. Sensitivity analyses were performed for outcomes with significant heterogeneity. The results indicated that the pooled effects for TT, androstenedione, DHEAS, and FG were significantly influenced by individual studies, suggesting that these findings should be interpreted with caution. For triglycerides and WHR, which involved only two meta-analyses each, sensitivity analysis showed consistent effect directions across individual studies, indicating that conclusions were not overly dependent on any single study. The reliability of the remaining outcomes was supported by sensitivity analyses, which confirmed that the findings remained consistent and were not disproportionately influenced by any single study.

### 3.8 Effects in cross-subgroup analysis of inositol subtypes versus control type

To elucidate the interaction effects between distinct inositol subtypes and control interventions, and to eliminate potential confounding variables, a cross-subgroup analysis was performed for all clinical outcomes in this study. Based on the types of intervention groups included in the studies, the inositol used in this article is categorized as follows:MI/MI+FA, DCI, (MI/MI+FA)+DCI, (MI/MI+FA) or DCI, 1.MI/MI+FA 2.(MI/MI+FA)+DCI, 1.MI/MI+FA 2.DCI 3.(MI/MI+FA)+DCI. Comprehensive results are provided in the **Supplementary Table 3**.

In the subgroup analysis of MI/MI+FA (15–23, 25), this subtype exerted significant beneficial effects on multiple biochemical and clinical indicators. Specifically, relative to the placebo, FA, or no-treatment controls, statistically significant reductions were observed in:FT(MD:-0.02, 95% CI: [-0.03, -0.00], *P* = 0.009, GRADE: Low); AUC Glucose(MD:-30.58, 95% CI:[-61.16, -0.00], *P* = 0.05, GRADE: Low); AUC Insulin(MD:-14126.48, 95% CI: [-18795.13, -9457.83], *P*<0.00001, GRADE: Low)In contrast, when compared with MET, only triglyceride levels exhibited a significant decrease(MD:-0.06, 95% CI: [-0.09, -0.02], *P=*0.0001, GRADE: Moderate).

Regarding reproductive outcomes, the pregnancy rate was significantly elevated in both the overall analysis(MD:1.31, 95% CI: [1.08, 1.60], *P* = 0.007, I^2^ = 40%, *P*-heterogeneity = 0.08, GRADE: Low) and the head-to-head comparisons with MET and DCI(vs. MET: MD:1.43, 95% CI: [1.14, 1.79], P = 0.0002, I^2^ = 0%, *P*-heterogeneity = 0.77, GRADE: Moderate; vs. DCI: MD:2.86, 95% CI: [1.14, 7.17], *P* = 0.03, GRADE: Moderate). Additionally, the ovulation rate was significantly increased relative to placebo and other control groups(MD:1.33, 95% CI: [0.99, 1.77], *P* = 0.05, I^2^ = 0%, *P*-heterogeneity = 0.73, GRADE: Moderate).

The significant therapeutic effects of the DCI (17) (24) subtype were restricted to comparisons with placebo or FA controls, with the following outcomes demonstrating statistical significance:TT, FT, DHEAS, SHBG, ovulation rate. However, only one study is included, the findings should be interpreted with caution.

For the combined (MI/MI+FA) or DCI subtype (14, 18), relative to placebo/FA controls, significant improvements were noted in key endocrine and metabolic parameters. Specifically, LH(MD:-3.50, 95% CI: [-4.89, -2.11], *P* = 0.001, GRADE: Low) and FSH(MD:-1.40, 95% CI: [-1.64, -1.16], *P* = 0.001, GRADE: Low) levels were markedly reduced; concentrations of TT, FT, and other sex steroids also decreased significantly, whereas SHBG levels were significantly elevated (MD:39.29, 95% CI: [30.25, 48.32], *P*<0.00001, I^2^ = 0%, *P*-heterogeneity = 0.82, GRADE: Moderate). Among metabolic indicators, FI, HOMA-IR, and AUC Insulin were significantly lowered. Furthermore, the pregnancy rate was significantly increased in this subgroup(RR:2.85, 95% CI: [1.30, 6.25], *P* = 0.009, I^2^ = 0, *P*-heterogeneity =0.88, GRADE: Moderate).

In the pooled analysis of MI/MI+FA and (MI/MI+FA)+DCI (19, 26), significant improvements were observed in LH(MD:-3.38, 95% CI: [-4.48, -2.28], *P* < 0.00001, GRADE: Moderate), FSH(MD:–0.89, 95% CI: [-1.54, -0.24], *P* = 0.008, GRADE: Moderate), FT(MD:-0.01, 95% CI: [-0.03, -0.00], *P* = 0.04, GRADE: Moderate), and SHBG levels. FI levels were significantly reduced relative to placebo/FA, OCP, and overall control groups, with concomitant decreases in HOMA-IR. All statistically significant indicators were assigned a moderate GRADE quality of evidence rating.

Finally, in the pooled analysis incorporating MI/MI+FA, DCI, and (MI/MI+FA)+DCI (17), relative to placebo/FA, significant reductions were detected in TT, FT, and androstenedione, along with a significant elevation in SHBG levels. Moreover, AUC Insulin and BMI were significantly decreased. All results in this analysis were rated as low-grade evidence quality and included only one study; outcomes should be interpreted with caution.

## 4 Discussion

### 4.1 Principal findings and possible explanations

In summary, our integrated analysis indicates that inositol significantly improves core hormonal, metabolic, and reproductive outcomes in PCOS patients. To establish this comprehensive evidence base, we systematically integrated 13 meta-analyses encompassing 85 outcome measures, concurrently evaluating both the methodological quality and the certainty of the evidence.Results indicate that inositol supplementation significantly improves multiple clinical indicators, including specific serum hormone levels, glucose metabolism-related parameters (e.g., FI, HOMA-IR, insulin AUC), lipid parameters (cholesterol and triglycerides), anthropometric measurements (WHR), and reproductive outcomes (pregnancy rate, live birth rate, and ovulation rate). This interpretation is based on evidence that varies in quality, with moderate certainty supporting certain key improvements despite the generally limited methodological rigor of the included studies. It is noteworthy that in the comprehensive statistical analysis, certain indicators did not demonstrate statistical significance. However, in subgroup analyses, these indicators exhibited significant differences, particularly within the control subgroups receiving either placebo or folic acid treatment. Compared to MET, inositol showed significant advantages in reducing triglycerides and improving pregnancy rates, while differences between the two interventions were not statistically significant for most other measures.

Approximately one-fourth of the included meta-analyses were of high methodological quality; however, none of the outcome measures demonstrated high-quality evidence, with specific details as follows:moderate (18.9%), low (40%), and very low (41.1%). Significant heterogeneity was observed in approximately 24.7% (21/85) of the outcomes. Despite comprehensive sensitivity analyses, heterogeneity persisted and could not be eliminated by removing any single study. This suggests that the heterogeneity stems from systematic differences between studies rather than from outliers. Several outcomes showed high heterogeneity, which may be attributable to variations in intervention type, dosage and duration, comparator groups (placebo, FA, MET, etc.), study design, outcome measurement, and clinical diversity in PCOS phenotypes. Subgroup analyses indicated that the type of control group was the primary contributor to heterogeneity.

To further investigate the relationship with intervention type, we conducted a re-subgroup analysis, re-stratifying according to intervention type and performing a cross-stratified analysis with the control group.Additionally, cross-subgroup analyses comparing the efficacy of different inositol isomers (such as MI, DCI, and their combinations) against various control groups (e.g., placebo/FA, MET) revealed distinct mechanisms of action. For instance, MI monotherapy demonstrated superior efficacy in improving metabolic and reproductive outcomes, while DCI monotherapy significantly reduced testosterone and androstenedione levels more effectively than the placebo/FA group. Notably, MI+DCI combination therapy did not consistently outperform monomer therapy across multiple outcome measures. However, some categories (DCI, MI+DCI) included only 1–2 studies, and the results should be interpreted with caution.

Although most outcomes exhibit high heterogeneity, we still report pooled effects to present overall trends, but their interpretation requires extreme caution.

Inositol, a naturally occurring carbohydrate of the sugar alcohol class, contributes to glucose metabolism in the human body. It is abundant in various health foods such as grains and seeds; additionally, the human body can synthesize approximately 4 grams daily through endogenous pathways (primarily in the kidneys). Among the nine stereoisomers of inositol, MI and DCI exhibit the most widespread natural distribution (27).

In mammalian systems, MI is widely distributed as the primary myo-inositol isomer, exhibiting extensive distribution across multiple tissues. It accumulates at significantly high concentrations in specific organs such as brain tissue, blood, adipose tissue, kidneys, lungs, ovaries, and testes. This compound participates in regulating multiple cellular metabolic pathways (9). And It has garnered significant attention owing to its potential benefits in fertility treatment. DCI, as an aromatase transcription inhibitor (aromatase being an enzyme that catalyzes the conversion of androgens into estrogens) (28), directly enhances testosterone biosynthesis in theca cells by downregulating aromatase activity in granulosa cells. Simultaneously, it reduces the conversion of testosterone into estradiol, thereby promoting androgen accumulation (29).

Inositol functions as a mediator in key physiological processes including energy metabolism, cell motility, and the development of ovarian follicles throughout the menstrual cycle. The maintenance of optimal glucose levels and their associated metabolic pathways plays a vital role in supporting both follicular maturation and overall female fertility (30), facilitating essential cell movements during embryonic development (e.g., neural tube closure) (31) and maintaining normal pregnancy. Furthermore, inositol participates in insulin signaling as a second messenger; current research indicates that inositol supplementation improves insulin sensitivity, metabolic markers, and spontaneous pregnancy rates in patients with PCOS (26).

Insulin resistance is considered a core pathophysiological mechanism of PCOS. This condition leads to hyperinsulinemia, which acts as a second messenger to elevate LH levels and increase granulosa cell sensitivity to LH. This ultimately triggers hyperandrogenism and ovulatory dysfunction while simultaneously reducing plasma SHBG levels, further elevating free androgen concentrations (32), creating a vicious cycle.

In healthy females, the plasma ratio of MI to DCI is approximately 40:1 (33); in follicular fluid, this ratio is even higher, typically maintained around 100:1 (34). Normal ovaries maintain this MI/DCI ratio within the physiological range of approximately 100:1 by regulating insulin-dependent isomerase activity. However, in women with PCOS, this ratio is significantly reversed, sometimes dropping as low as 0.2:1 (35). This metabolic imbalance simultaneously elevates DCI levels, promoting androgen synthesis (36); while severe MI depletion simultaneously disrupts FSH signaling and degrades oocyte quality. MI enhances insulin sensitivity, aiding PCOS patients in restoring ovulation and hormonal balance (37). Therefore, correcting the disrupted MI/DCI ratio towards physiological levels is the objective of inositol supplementation in PCOS, for which a 40:1 combination therapy serves as an effective intervention to enhance insulin sensitivity (38). By regulating the reproductive axis and improving metabolic parameters, inositol treatment has been shown to enhance ovulation and ovarian function, reduce hyperandrogenism and its clinical manifestations (such as acne and hirsutism), and ultimately support fertility (39).

Additional research confirms that MI’s role in improving PCOS manifestations may be linked to its mitigation of underlying inflammatory states (40). However, current evidence remains insufficient to support incorporating inositol supplementation into guideline-recommended standard treatment protocols.

This study conducted a multidimensional analysis of inositol’s effects on PCOS patients, examining indicators including hormone levels, glucose metabolism, lipid profiles, anthropometric measurements, and reproductive outcomes. Subgroup analyses were performed for most indicators based on control group type (Place/FA, MET). Results indicate that inositol significantly improves PCOS patients’ LH, FSH, TT, FT, androstenedione, SHBG, and DHEAS levels, with more pronounced effects observed in the placebo/FA group.

Concurrently, as an insulin sensitizer, MI exerted beneficial effects on metabolic disorders. Beyond regulating insulin resistance and aiding in the adjustment of FI, HOMA-IR, glucose AUC, and insulin AUC, it reduced body weight and positively influenced patients’ return to normal BMI and WHR. Furthermore, it demonstrates efficacy in lowering blood lipids. This study found improvements in cholesterol and triglycerides among PCOS patients following inositol treatment. A separate meta-analysis focusing on lipid metabolism reported a differential effect of inositol: it elevated HDL cholesterol levels specifically in PCOS patients, with no significant change observed in non-PCOS individuals (41). In PCOS patients, disrupted inositol ratios impair oocyte quality, subsequently affecting ovulation and embryo quality. This study found that inositol supplementation increases pregnancy rates, live birth rates, and ovulation rates. In recent years, inositol has also been actively used in pre-reproductive assistance treatment for PCOS patients to enhance assisted reproductive success rates and reduce side effects such as ovarian hyperstimulation syndrome (42–44). In terms of safety, compared to MET, which commonly causes gastrointestinal adverse reactions, inositol demonstrates a lower risk of adverse events. This makes inositol a potentially safer alternative treatment option for patients who cannot tolerate adequate doses of MET (16).

Furthermore, we conducted subgroup analyses based on different inositol subtypes (such as MI, DCI, and their combinations) to further explore differences in efficacy among these subtypes.These findings suggest that the optimal inositol formulation may depend on specific clinical objectives—whether hormone regulation, metabolic improvement, or reproductive function optimization. They underscore that inositol supplementation protocols should be tailored not only to patient phenotypes but also to therapeutic goals, highlighting the necessity for future studies to directly compare isomer-specific effects within standardized trial designs. Based on the above findings, the following recommendations can guide clinical practice: For instance, the MI/MI+FA regimen is more suitable for PCOS patients with insulin resistance and fertility needs. Subgroup analysis indicates that DCI monotherapy may reduce relevant androgen levels, yet this conclusion is based exclusively on a single meta-analysis. Given prior evidence that DCI monotherapy suppresses aromatase synthesis and promotes androgen production, thereby worsening hyperandrogenism in PCOS, the clinical application of DCI monotherapy should be carefully evaluated. In this study, combination therapy with MI and DCI showed no synergistic advantages, and its efficacy was on par with that of MI monotherapy.

It must be emphasized that the evidence quality for some results was rated as low or very low. Interpretation of these positive effects requires extreme caution. The low quality stems from small study sizes and heterogeneity. While this finding suggests potential value of inositol in improving these parameters, it should be regarded as a preliminary signal rather than a definitive conclusion.

### 4.2 Strengths and limitations

This study possesses multiple significant advantages. It systematically integrates currently available research evidence, comprehensively revealing the multifaceted health impacts of inositol on women with PCOS across reproductive endocrinology, glucose metabolism, lipid profiles, and body composition. This study is the first to comprehensively compare the efficacy differences of MI, DCI, and combined MI+DCI therapy across various clinical endpoints in PCOS patients using an umbrella analysis, providing evidence to further elucidate the functional characteristics of different inositol subtypes. Moreover, this study represents the first panoramic assessment and systematic review of existing meta-analytic evidence on inositol treatment for PCOS. It not only expands perspectives on current clinical treatment protocols and provides evidence-based supplementation but also establishes a solid reference foundation for determining future research directions and in-depth exploration in this field.

However, this study also exhibits several methodological limitations warranting attention: The limited number of original studies included in the meta-analysis may limit the statistical power and reliability of the synthesized findings. Additionally, most interventions lasted only 3 to 24 weeks, lacking long-term follow-up data exceeding one year. Consequently, it remains impossible to assess the impact of inositol supplementation on long-term health outcomes in PCOS patients, such as cardiovascular disease risk and diabetes progression. Furthermore, most subgroup analyses exhibited significant heterogeneity. Due to fewer than 10 original studies per outcome measure, small-sample effect analyses like funnel plot testing were unfeasible, leading to downgraded evidence quality assessments. For certain indicators (e.g., LH, FSH, and lipid parameters), support was based on only two studies, limiting sensitivity analysis capabilities to identify sources of bias and hindering further subgroup analyses exploring heterogeneity causes. Although our cross-subgroup analyses provided valuable insights into the differential effects of inositol isomers across control types, these comparisons were often based on limited studies and exhibited substantial heterogeneity, which precludes definitive conclusions regarding the superiority of any specific isomer or combination. Finally, the studies did not formally evaluate the overlap of original randomized controlled trials across different meta-analyses, potentially introducing a certain risk of duplicate bias.

### 4.3 Clinical implications

Based on the aforementioned empirical research, this study proposes the following clinical application recommendations:

- Recommended Dosage: Based on effective doses reported in the majority of included studies, a daily intake of 2-4g of inositol is suggested for women with PCOS, preferably in combination with folic acid (e.g., MI+FA), as subgroup analysis indicates that this combination therapy yields more pronounced efficacy.
- Applicable population: Considering subgroup results compared to MET, it is proposed that for PCOS patients intolerant to MET (e.g., due to gastrointestinal reactions) or with clear pregnancy planning needs, inositol may serve as an alternative or adjunctive therapy, particularly demonstrating superiority over MET in improving pregnancy rates.
- Treatment duration recommendation: Based on intervention duration data, it is recommended to supplement continuously for at least 12 weeks to observe stable improvements in hormonal and metabolic markers (most high-quality studies followed up for ≥12 weeks).

### 4.4 Future perspectives

These findings may offer novel insights for clinical PCOS management: Inositol could serve as a primary or adjunctive therapeutic agent. While this discovery suggests potential value in improving these parameters, it should be regarded as a preliminary signal rather than a definitive conclusion. The significance of these findings lies in guiding future clinical research. Therefore, it is strongly recommended to conduct more rigorously designed, adequately powered, multicenter RCTs with standardized protocols to address current heterogeneity issues, thereby providing more reliable and higher-level evidence for the efficacy of inositol. Further attention should be given to establishing clear direct comparisons between inositol isomers (MI and DCI) and their synergistic effects with other interventions, as well as their long-term impact on PCOS-related complications. Additionally, priority should be given to collecting long-term health data on MI treatment for PCOS.

## 5 Conclusions

This study demonstrates that inositol treatment shows particular efficacy in improving hormonal parameters (including TT, FT, and SHBG), enhancing insulin sensitivity (as measured by HOMA-IR), and improving key reproductive outcomes (pregnancy and ovulation rates). Moderate-quality GRADE evidence supporting the aforementioned efficacy provides a reasonable basis for considering inositol as a viable treatment option in clinical practice. Cross-subgroup analysis further reveals differential effects of inositol subtypes: MI/MI+FA is more suitable for PCOS patients with insulin resistance and fertility needs, while DCI monotherapy should be used with caution in clinical practice. These findings suggest that inositol represents a promising alternative, particularly for patients who are intolerant to metformin or have fertility needs. However, outcomes supported by low or very low-quality evidence, such as FSH levels and glucose area under curve, require cautious interpretation and should not form the basis of standalone clinical decisions.Therefore, these findings require confirmation through large-scale, more rigorous randomized controlled trials to establish the precise therapeutic effects of inositol.

## Data Availability

The original contributions presented in the study are included in the article/**Supplementary Material**. Further inquiries can be directed to the corresponding author.
