# Supplementary material for: Effects of inositol in women with polycystic ovary syndrome: an umbrella review of meta-analyses from randomized controlled trials
Source: Front Endocrinol (Lausanne). 2026 Feb 11;17:1741509. doi: 10.3389/fendo.2026.1741509 (PMC12932251; doi:10.3389/fendo.2026.1741509)
Supplement: Supplementary file 2 [file Table2.docx]

**Supplementary Table 2 Search Strategy**

1. **Pubmed**

| #1 ((((Polycystic Ovary Syndrome[MeSH Terms]) ) )) OR (Ovary Syndrome, Polycystic OR Syndrome, Polycystic Ovary OR Polycystic Ovarian Syndrome OR Ovarian Syndrome, Polycystic OR Polycystic Ovary Syndrome 1 OR Sclerocystic Ovarian Degeneration OR Ovarian Degeneration, Sclerocystic OR Sclerocystic Ovary Syndrome OR Stein-Leventhal Syndrome OR Stein Leventhal Syndrome OR Syndrome, Stein-Leventhal OR Sclerocystic Ovaries OR Ovary, Sclerocystic OR Sclerocystic Ovary) |
| --- |
| #2 (Inositol[MeSH Terms]) OR (Chiro-Inositol[Title/Abstract] OR Chiro Inositol[Title/Abstract] OR Mesoinositol[Title/Abstract] OR Myoinositol[Title/Abstract]) |
| #3 (((Meta-Analysis as Topic[MeSH Terms]) OR (Systematic Reviews as Topic[MeSH Terms])) OR (Meta-Analysis[Title/Abstract] OR meta-analyses[Title/Abstract] OR meta-analyze[Title/Abstract] OR Systematic Review[Title/Abstract])) OR (Meta-Analysis[Publication Type] OR Systematic Review[Publication Type]) |
| #4 #1 AND #2 AND #3 |

1. **Embase**

| #1 'ovary polycystic disease'/exp OR 'ovary polycystic disease' |
| --- |
| #2 'cystic ovary':ab,kw,ti OR 'micropolycystic ovary':ab,kw,ti OR 'multiple follicle cyst':ab,kw,ti OR 'ovary polycystic syndrome':ab,kw,ti OR 'ovary, micropolycystic':ab,kw,ti OR 'ovary, polycystic':ab,kw,ti OR 'polycystic ovarian disease':ab,kw,ti OR 'polycystic ovary':ab,kw,ti OR 'polycystic ovary disease':ab,kw,ti OR 'polycystic ovary syndrome':ab,kw,ti OR 'stein cohen leventhal syndrome':ab,kw,ti OR 'stein leventhal disease':ab,kw,ti OR 'stein leventhal syndrome':ab,kw,ti OR 'syndrome stein leventhal':ab,kw,ti OR 'ovary polycystic disease':ab,kw,ti |
| #3 #1 OR #2 |
| #4 'inositol'/exp OR 'inositol' |
| #5 'betitol':ab,kw,ti OR 'bios i':ab,kw,ti OR 'cyclohexane 1, 2, 3, 4, 5, 6 hexol':ab,kw,ti OR 'cyclohexanehexol':ab,kw,ti OR 'cyclohexitol':ab,kw,ti OR 'dambose':ab,kw,ti OR 'hexol':ab,kw,ti OR 'i inositol':ab,kw,ti OR 'inosil':ab,kw,ti OR 'inosimesol':ab,kw,ti OR 'inosit':ab,kw,ti OR 'inosite':ab,kw,ti OR 'inositene':ab,kw,ti OR 'inositina':ab,kw,ti OR 'inositol medium':ab,kw,ti OR 'm inositol':ab,kw,ti OR 'meat sugar':ab,kw,ti OR 'mesitol':ab,kw,ti OR 'meso 1, 2, 3, 4, 5, 6 hexahydroxycyclohexane':ab,kw,ti OR 'meso inositol':ab,kw,ti OR 'mesoinosil':ab,kw,ti OR 'mesoinosite':ab,kw,ti OR 'mesoinositol':ab,kw,ti OR 'mesol':ab,kw,ti OR 'mesovil':ab,kw,ti OR 'mouse antialopecia factor':ab,kw,ti OR 'muscle sugar':ab,kw,ti OR 'myo inositol':ab,kw,ti OR 'myoinosil':ab,kw,ti OR 'myoinositol':ab,kw,ti OR 'nucite':ab,kw,ti OR 'phaseomannite':ab,kw,ti OR 'phaseomannitol':ab,kw,ti OR 'rat antispectacled eye factor':ab,kw,ti OR 'scyllite':ab,kw,ti OR 'tonozil':ab,kw,ti OR 'inositol':ab,kw,ti |
| #6 #4 OR #5 |
| #7 'meta analysis'/exp OR 'meta analysis' |
| #8 'analysis, meta':ab,kw,ti OR 'meta-analysis':ab,kw,ti OR 'meta analysis'/exp OR 'meta analysis' OR 'meta analysis':ab,kw,ti |
| #9 #7 OR #8 |
| #10 'systematic review (topic)'/exp OR 'systematic review (topic)' |
| #11 'systematic reviews':ab,kw,ti OR 'systematic reviews as topic':ab,kw,ti OR 'systematic review (topic)':ab,kw,ti |
| #12 #10 OR #11 |
| #13 #9 OR #12 |
| #14 #3 AND #6 AND #13 |

1. ****Web of Science****

| **#1 Polycystic Ovary Syndrome OR Ovary Syndrome, Polycystic OR Syndrome, Polycystic Ovary OR Polycystic Ovarian Syndrome OR Ovarian Syndrome, Polycystic OR Polycystic Ovary Syndrome 1 OR Sclerocystic Ovarian Degeneration OR Ovarian Degeneration, Sclerocystic OR Sclerocystic Ovary Syndrome OR Stein-Leventhal Syndrome OR Stein Leventhal Syndrome OR Syndrome, Stein-Leventhal OR Sclerocystic Ovaries OR Ovary, Sclerocystic OR Sclerocystic Ovary (Topic)** |
| --- |
| **#2 Inositol OR Chiro-Inositol OR Chiro Inositol OR Mesoinositol OR Myoinositol (Topic)** |
| **#3 Meta-Analysis as Topic OR Systematic Reviews as Topic OR Meta-Analysis OR meta-analyses OR Meta-Analysis OR meta-analyze OR Systematic Review (Topic)** |
| **#4 #1 AND #2 AND #3** |

1. ****Scopus****

| **#1 TITLE-ABS-KEY ( Polycystic Ovary Syndrome ) OR TITLE-ABS-KEY ( Ovary Syndrome , Polycystic ) OR TITLE-ABS-KEY ( Syndrome , Polycystic Ovary ) OR TITLE-ABS-KEY ( Polycystic Ovarian Syndrome ) OR TITLE-ABS-KEY ( Ovarian Syndrome , Polycystic ) OR TITLE-ABS-KEY ( Polycystic Ovary Syndrome 1 ) OR TITLE-ABS-KEY ( Sclerocystic Ovarian Degeneration ) OR TITLE-ABS-KEY ( Ovarian Degeneration , Sclerocystic ) OR TITLE-ABS-KEY ( Sclerocystic Ovary Syndrome ) OR TITLE-ABS-KEY ( Stein-Leventhal Syndrome ) OR TITLE-ABS-KEY ( Stein Leventhal Syndrome OR Syndrome , Stein-Leventhal ) OR TITLE-ABS-KEY ( Sclerocystic Ovaries ) OR TITLE-ABS-KEY ( Ovary , Sclerocystic ) OR TITLE-ABS-KEY ( Sclerocystic Ovary )** |
| --- |
| **#2 TITLE-ABS-KEY ( Inositol ) OR TITLE-ABS-KEY ( Chiro-Inositol ) OR TITLE-ABS-KEY ( Chiro Inositol ) OR TITLE-ABS-KEY ( Mesoinositol ) OR TITLE-ABS-KEY ( Myoinositol )** |
| **#3 TITLE-ABS-KEY ( Meta-Analysis ) OR TITLE-ABS-KEY ( meta-analyses ) OR TITLE-ABS-KEY ( Meta-Analysis ) OR TITLE-ABS-KEY ( meta-analyze ) OR TITLE-ABS-KEY ( Systematic Review )** |
| **#4 #1 AND #2 AND #3** |
