# Supplementary material for: Effects of inositol in women with polycystic ovary syndrome: an umbrella review of meta-analyses from randomized controlled trials
Source: Front Endocrinol (Lausanne). 2026 Feb 11;17:1741509. doi: 10.3389/fendo.2026.1741509 (PMC12932251; doi:10.3389/fendo.2026.1741509)
Supplement: Supplementary file 3 [file Table3.doc]

*Supplementary Table 3 The effects in cross-subgroup analysis of inositol subtypes versus control type*

| **Outcomes stratified by different covariates** | **No.  studies** | **Intervention** | **Control** | **Pooled effect size(WD/RR)**  **(95% CI)** | ***P*-value** | **I^2^**  **(%)** | ***P*-heterogeneity** | **Evidence certainty**  **(GRADEError: Reference source not found)** |
| --- | --- | --- | --- | --- | --- | --- | --- | --- |
| MI/MI+FA | | | | | | | | |
| TT (nmol/L) | 3 | MI/MI+FA |  | 0.01 [-0.12, 0.13] | 0.91 | 0 | 0.62 | Low |
|  | 1 | MI/MI+FA | Placebo/FA/No treatment | -0.40 [-1.02, 0.23] | 0.22 | / | / | Very low |
|  | 3 | MI/MI+FA | MET | 0.02 [-0.10, 0.15] | 0.71 | 0 | 0.94 | Low |
| FT (nmol/L) | 1 | MI/MI+FA | Placebo/FA/No treatment | -0.02 [-0.03, -0.00] | 0.009 | / | / | Low |
| Androstenidione (nmol/L) | 2 | MI/MI+FA |  | -1.36 [-4.33, 1.60] | 0.37 | 84 | 0.01 | Very low |
|  | 1 | MI/MI+FA | Placebo/FA/No treatment | -3.11 [-5.45, -0.77] | 0.009 | / | / | Low |
|  | 1 | MI/MI+FA | MET | -0.05 [-0.65, 0.55] | 0.87 | / | / | Low |

*Continued*

| **Outcomes stratified by different covariates** | **No.  studies** | **Intervention** | **Control** | **Pooled effect size(WD/RR)**  **(95% CI)** | ***P*-value** | **I^2^**  **(%)** | ***P*-heterogeneity** | **Evidence certainty**  **(GRADEError: Reference source not found)** |
| --- | --- | --- | --- | --- | --- | --- | --- | --- |
| SHBG (nmol/L) | 3 | MI/MI+FA |  | -2.37 [-10.68, 5.93] | 0.58 | 71 | 0.01 | Very low |
|  | 1 | MI/MI+FA | Placebo/FA/No treatment | 37.60 [-43.97, 119.17] | 0.37 | / | / | Very low |
|  | 3 | MI/MI+FA | MET | -2.81 [-11.28, 5.65] | 0.51 | 79 | 0.008 | Very low |
| DDHEAS (µmol/L) | 1 | MI/MI+FA |  | -0.47 [-3.55, 2.61] | 0.77 | 49 | 0.16 | Very low |
|  | 1 | MI/MI+FA | Placebo/FA/No treatment | -3.09 [-7.99, 1.81] | 0.22 | / | / | Very low |
|  | 1 | MI/MI+FA | MET | 0.47 [-0.49, 1.43] | 0.34 | / | / | Very low |
| FG (mmol/l) | 2 | MI/MI+FA | MET | 0.13 [-2.52, 2.78] | 0.92 | 0 | 0.36 | Low |
| FI (pmol/L) | 3 | MI/MI+FA | MET | -0.45 [-1.73, 0.83] | 0.49 | 0 | 0.78 | Low |

*Continued*

| **Outcomes stratified by different covariates** | **No.  studies** | **Intervention** | **Control** | **Pooled effect size(WD/RR)**  **(95% CI)** | ***P*-value** | **I^2^**  **(%)** | ***P*-heterogeneity** | **Evidence certainty**  **(GRADEError: Reference source not found)** |
| --- | --- | --- | --- | --- | --- | --- | --- | --- |
|  |  |  |  |  |  |  |  |  |
| HOMA-IR | 4 | MI/MI+FA |  | -0.12 [-0.30, 0.06] | 0.19 | 54 | 0.07 | Very low |
|  | 1 | MI/MI+FA | Placebo/FA/No treatment | -0.17 [-1.57, 1.23] | 0.81 | / | / | Very low |
|  | 4 | MI/MI+FA | MET | -0.12 [-0.31, 0.08] | 0.24 | 66 | 0.03 | Very low |
| AUC Glucose (mmol・min/L) | 1 | MI/MI+FA |  | 2.92 [-88.34, 94.18] | 0.95 | 63 | 0.1 | Very low |
|  | 1 | MI/MI+FA | Placebo/FA/No treatment | -30.58 [-61.16, -0.00] | 0.05 | / | / | Low |
|  | 1 | MI/MI+FA | MET | 67.64 [-45.12, 180.40] | 0.24 | / | / | Very low |
| AUC insulin (pmol・min/L) | 1 | MI/MI+FA |  | -5984.35 [-28035.69, 16067.00] | 0.59 | 67 | 0.08 | Very low |

*Continued*

| **Outcomes stratified by different covariates** | **No.  studies** | **Intervention** | **Control** | **Pooled effect size(WD/RR)**  **(95% CI)** | ***P*-value** | **I^2^**  **(%)** | ***P*-heterogeneity** | **Evidence certainty**  **(GRADEError: Reference source not found)** |
| --- | --- | --- | --- | --- | --- | --- | --- | --- |
|  | 1 | MI/MI+FA | Placebo/FA/No treatment | -14126.48 [-18795.13, -9457.83] | ＜0.00001 | / | / | Low |
|  | 1 | MI/MI+FA | MET | 9562.26 [-16811.94, 35936.46] | 0.48 | / | / | Very low |
| Cholesterol (mmol/l) | 1 | MI/MI+FA | MET | 0.07 [-0.04, 0.17] | 0.28 | / | / | Low |
| Triglycerides (mmol/l) | 1 | MI/MI+FA | MET | -0.06 [-0.09, -0.02] | 0.0001 | / | / | Moderate |
| HDL(mmol/l) | 1 | MI/MI+FA | MET | -0.06 [-0.24, 0.11] | 0.76 | / | / | Low |
| LDL(mmol/l) | 1 | MI/MI+FA | MET | 0.08 [-0.07, 0.23] | 0.26 | / | / | Low |
| BMI(kg/m²) | 5 | MI/MI+FA |  | 0.00 [-0.40, 0.40] | 0.99 | 47 | 0.1 | Very low |
|  | 2 | MI/MI+FA | Placebo/FA/No treatment | -0.52 [-1.07, 0.04] | 0.07 | 0 | 0.4 | Low |

*Continued*

| **Outcomes stratified by different covariates** | **No.  studies** | **Intervention** | **Control** | **Pooled effect size(WD/RR)**  **(95% CI)** | ***P*-value** | **I^2^**  **(%)** | ***P*-heterogeneity** | **Evidence certainty**  **(GRADEError: Reference source not found)** |
| --- | --- | --- | --- | --- | --- | --- | --- | --- |
|  | 4 | MI/MI+FA | MET | 0.29 [-0.27, 0.86] | 0.31 | 52 | 0.1 | Very low |
| WHR | 1 | MI/MI+FA | MET | 0.02 [-0.02, 0.06] | 0.97 | / | / | Low |
| Pregnancy rates | 5 | MI/MI+FA |  | 1.31 [1.08, 1.60] | 0.007 | 40 | 0.08 | Low |
|  | 5 | MI/MI+FA | Placebo/FA/No treatment | 1.30 [0.95, 1.76] | 0.1 | 29 | 0.23 | Very low |
|  | 4 | MI/MI+FA | MET | 1.43 [1.14, 1.79] | 0.0002 | 0 | 0.77 | Moderate |
|  | 1 | MI/MI+FA | antioxidant (melatonin) | 0.88 [0.66, 1.18] | 0.39 | / | / | Low |
|  | 1 | MI/MI+FA | DCI | 2.86 [1.14, 7.17] | 0.03 | / | / | Moderate |
| Live births | 1 | MI/MI+FA |  | 1.41 [0.81, 2.48] | 0.23 | 0 | 0.36 | Moderate |
|  |  |  |  |  |  |  |  |  |

*Continued*

| **Outcomes stratified by different covariates** | **No.  studies** | **Intervention** | **Control** | **Pooled effect size(WD/RR)**  **(95% CI)** | ***P*-value** | **I^2^**  **(%)** | ***P*-heterogeneity** | **Evidence certainty**  **(GRADEError: Reference source not found)** |
| --- | --- | --- | --- | --- | --- | --- | --- | --- |
|  | 1 | MI/MI+FA | Placebo/FA/No treatment | 2.07 [0.77, 5.57] | 0.15 | / | / | Moderate |
|  | 1 | MI/MI+FA | antioxidant (melatonin) | 1.18 [0.60, 2.33] | 0.63 | / | / | Moderate |
| Ovulation RATE | 2 | MI/MI+FA |  | 1.11 [0.80, 1.55] | 0.54 | 56 | 0.1 | Low |
|  | 2 | MI/MI+FA | Placebo/FA/No treatment | 1.33 [0.99, 1.77] | 0.05 | 0 | 0.73 | Moderate |
|  | 1 | MI/MI+FA | antioxidant (melatonin) | 0.87 [0.67, 1.13] | 0.3 | / | / | Moderate |
|  |  |  |  |  |  |  |  |  |
|  |  |  |  |  |  |  |  |  |
|  |  |  |  |  |  |  |  |  |

*Continued*

| **Outcomes stratified by different covariates** | **No.  studies** | | **Intervention** | | **Control** | | **Pooled effect size(WD/RR)**  **(95% CI)** | ***P*-value** | | **I^2^**  **(%)** | ***P*-heterogeneity** | | | **Evidence certainty**  **(GRADEError: Reference source not found)** | |
| --- | --- | --- | --- | --- | --- | --- | --- | --- | --- | --- | --- | --- | --- | --- | --- |
| DCI | | | | | | | | | | | | | | | |
| TT(nmol/L) | 1 | DCI | | Placebo/FA | | -1.45 [-2.43, -0.47] | | | 0.004 | | | / | / | | Low |
| FT(nmol/L) | 1 | DCI | | Placebo/FA | | -0.02 [-0.03, -0.01] | | | 0.0002 | | | / | / | | Low |
| Androstenidione(nmol/L) | 1 | DCI | | Placebo/FA | | -1.81 [-3.63, -0.00] | | | 0.09 | | | / | / | | Low |
| SHBG (nmol/L) | 1 | DCI | | Placebo/FA | | 55.45 [25.99, 84.91] | | | 0.0002 | | | / | / | | Low |
| DHEAS (µmol/L) | 1 | DCI | | Placebo/FA | | -4.57 [-7.63, -1.51] | | | 0.003 | | | / | / | | Low |
| AUC Glucose (mmol・min/L) | 1 | DCI | | Placebo/FA | | -83.38 [-189.04, 22.28] | | | 0.12 | | | / | / | | Very low |
|  |  |  | |  | |  | | |  | | |  |  | |  |

*Continued*

| **Outcomes stratified by differentcovariates** | **No.  studies** | **Intervention** | **Control** | **Pooled effect size(WD/RR)**  **(95% CI)** | ***P*-value** | **I^2^**  **(%)** | ***P*-heterogeneity** | **Evidencecertainty (GRADEError: Reference source not found)** |
| --- | --- | --- | --- | --- | --- | --- | --- | --- |
| AUC insulin (pmol・min/L) | 1 | DCI | Placebo/FA | -27968.70 [-58008.85, 2071.45] | 0.06 | / | / | Very low |
| BMI(kg/m²) | 1 | DCI | Placebo/FA | 0.35 [-0.57, 1.27] | 0.44 | / | / | Very low |
| Ovulation rate | 1 | DCI | Placebo/FA | 1.16 [1.07, 1.26] | 0.0006 | / | / | Moderate |
|  |  |  |  |  |  |  |  |  |
|  |  |  |  |  |  |  |  |  |
|  |  |  |  |  |  |  |  |  |
|  |  |  |  |  |  |  |  |  |
|  |  |  |  |  |  |  |  |  |
|  |  |  |  |  |  |  |  |  |

*Continued*

| **Outcomes stratified by differentcovariates** | **No.  studies** | **Intervention** | **Control** | **Pooled effect size(WD/RR)**  **(95% CI)** | ***P*-value** | **I^2^**  **(%)** | ***P*-heterogeneity** | **Evidencecertainty (GRADE^1^)** |
| --- | --- | --- | --- | --- | --- | --- | --- | --- |
| (MI/MI+FA) +DCI | | | | | | | | |
| FT(nmol/L) | 1 | (MI/MI+FA) +DCI | Placebo/FA | -0.00 [-0.01, 0.00] | / | / | / | Very low |
| Androstenidione (nmol/L) | 1 | (MI/MI+FA) +DCI | Placebo/FA | 0.42 [-4.54, 5.38] | / | / | / | Very low |
| SHBG(nmol/L) | 1 | (MI/MI+FA) +DCI | Placebo/FA | 10.82 [-1.70, 23.34] | / | / | / | Very low |
| DHEAS (µmol/L) | 1 | (MI/MI+FA) +DCI | Placebo/FA | 1.15 [-2.43, 4.74] | / | / | / | Very low |
| HOMA-IR | 1 | (MI/MI+FA) +DCI | Placebo/FA | -0.73 [-2.20, 0.74] | / | / | / | Very low |
| BMI(kg/m²) | 1 | (MI/MI+FA) +DCI | Placebo/FA | -0.21 [-4.44, 4.02] | / | / | / | Very low |
|  |  |  |  |  |  |  |  |  |

*Continued*

| **Outcomes stratified by differentcovariates** | **No.  studies** | **Intervention** | **Control** | **Pooled effect size(WD/RR)**  **(95% CI)** | ***P*-value** | **I^2^**  **(%)** | ***P*-heterogeneity** | **Evidencecertainty (GRADE^1^)** |
| --- | --- | --- | --- | --- | --- | --- | --- | --- |
| Pregnancy rates/Clinical pregnancy | 1 | (MI/MI+FA) +DCI | Placebo/FA | 1.45 [1.06, 1.98] | / | / | / | Low |
| FT | 1 | (MI/MI+FA) +DCI | Placebo/FA | -0.00 [-0.01, 0.00] | / | / | / | Very low |
|  |  |  |  |  |  |  |  |  |
|  |  |  |  |  |  |  |  |  |
|  |  |  |  |  |  |  |  |  |
|  |  |  |  |  |  |  |  |  |
|  |  |  |  |  |  |  |  |  |
|  |  |  |  |  |  |  |  |  |
|  |  |  |  |  |  |  |  |  |

*Continued*

| **Outcomes stratified by differentcovariates** | **No.  studies** | **Intervention** | **Control** | **Pooled effect size(WD/RR)**  **(95% CI)** | ***P*-value** | **I^2^**  **(%)** | ***P*-heterogeneity** | **Evidencecertainty (GRADE^1^)** |
| --- | --- | --- | --- | --- | --- | --- | --- | --- |
| (MI/MI+FA) or DCI | | | | | | | | |
| LH (IU/L) | 1 | (MI/MI+FA) or DCI | Placebo/FA | -3.50 [-4.89, -2.11] | 0.001 | / | / | Low |
| FSH (IU/L) | 1 | (MI/MI+FA) or DCI | Placebo/FA | -1.40 [-1.64, -1.16] | 0.001 | / | / | Low |
| TT (nmol/L) | 1 | (MI/MI+FA) or DCI | Placebo/FA | -1.06 [-2.18, 0.06] | 0.0004 | / | / | Low |
| FT (nmol/L) | 2 | (MI/MI+FA) or DCI | Placebo/FA | -0.02 [-0.02, -0.02] | ＜0.00001 | 0 | 1 | Moderate |
| Androstenidione (nmol/L) | 2 | (MI/MI+FA) or DCI | Placebo/FA | -2.96 [-4.33, -1.59] | ＜0.0001 | 61 | 0.11 | Low |
| SHBG (nmol/L) | 2 | (MI/MI+FA) or DCI | Placebo/FA | 39.29 [30.25, 48.32] | ＜0.00001 | 0 | 0.82 | Moderate |
| DHEAS(µmol/L) | 1 | (MI/MI+FA) or DCI | Placebo/FA | -3.60 [-4.01, -3.19] | 0.02 | / | / | Moderate |

*Continued*

| **Outcomes stratified by differentcovariates** | **No.  studies** | **Intervention** | **Control** | **Pooled effect size(WD/RR)**  **(95% CI)** | ***P*-value** | **I^2^**  **(%)** | ***P*-heterogeneity** | **Evidencecertainty (GRADE^1^)** |
| --- | --- | --- | --- | --- | --- | --- | --- | --- |
| FG (mmol/l) | 2 | (MI/MI+FA) or DCI | Placebo/FA | -1.96 [-8.28, 4.37] | 0.54 | 96 | ＜0.00001 | Vrey low |
| FI (pmol/L) | 2 | (MI/MI+FA) or DCI | Placebo/FA | -23.53 [-38.62, -8.44] | 0.002 | 84 | 0.01 | Low |
| HOMA-IR | 2 | (MI/MI+FA) or DCI | Placebo/FA | -1.20 [-1.48, -0.91] | ＜0.00001 | 17 | 0.27 | Moderate |
| AUC Glucose (mmol・min/L) | 1 | (MI/MI+FA) or DCI | Placebo/FA | -139.62 [-157.28, -121.96] | 0.06 | / | / | Moderate |
| AUC insulin (pmol・min/L) | 1 | (MI/MI+FA) or DCI | Placebo/FA | -24200.00 [-29295.91, -19104.09] | < 0.00001 | / | / | Moderate |
| Pregnancy rates/Clinical pregnancy | 2 | (MI/MI+FA) or DCI | Placebo/FA | 2.85 [1.30, 6.25] | 0.009 | 0 | 0.88 | Moderate |
| Live birth | 1 | (MI/MI+FA) or DCI | Placebo/FA | 2.67 [0.80, 8.91] | 0.11 | / | / | Low |

*Continued*

| **Outcomes stratified by differentcovariates** | **No.  studies** | **Intervention** | **Control** | **Pooled effect size(WD/RR)**  **(95% CI)** | ***P*-value** | **I^2^**  **(%)** | ***P*-heterogeneity** | **Evidencecertainty (GRADE^1^)** |
| --- | --- | --- | --- | --- | --- | --- | --- | --- |
| 1.MI/MI+FA 2.(MI/MI+FA)+DCI | | | | | | | | |
| LH(IU/L) | 1 | 1.MI/MI+FA 2.(MI/MI+FA)+DCI | Placebo/FA | -3.38 [-4.48, -2.28] | < 0.00001 | / | / | Moderate |
| FSH(IU/L) | 1 | 1.MI/MI+FA 2.(MI/MI+FA)+DCI | Placebo/FA | -0.89 [-1.54, -0.24] | 0.008 | / | / | Moderate |
| TT(nmol/L ) | 1 | 1.MI/MI+FA 2.(MI/MI+FA)+DCI | Placebo/FA | -0.98 [-2.31, 0.35] | 0.15 | / | / | Low |
| FT(nmol/L ) | 1 | 1.MI/MI+FA 2.(MI/MI+FA)+DCI | Placebo/FA | -0.01 [-0.03, -0.00] | 0.04 | / | / | Moderate |
| Androstenidione（nmol/l） | 1 | 1.MI/MI+FA 2.(MI/MI+FA)+DCI | Placebo/FA | -1.50 [-3.21, 0.21] | 0.08 | / | / | Low |
| SHBG(nmol/L ) | 1 | 1.MI/MI+FA 2.(MI/MI+FA)+DCI | Placebo/FA | 24.01 [3.96, 44.06] | 0.02 | / | / | Moderate |

*Continued*

| **Outcomes stratified by differentcovariates** | **No.  studies** | **Intervention** | **Control** | **Pooled effect size(WD/RR)**  **(95% CI)** | ***P*-value** | **I^2^**  **(%)** | ***P*-heterogeneity** | **Evidencecertainty (GRADE^1^)** |
| --- | --- | --- | --- | --- | --- | --- | --- | --- |
| DHEAS(µmol/L) | 1 | 1.MI/MI+FA 2.(MI/MI+FA)+DCI | Placebo/FA | -1.54 [-5.89, 2.81] | 0.49 | / | / | Low |
| FG (mmol/l) | 1 | 1.MI/MI+FA 2.(MI/MI+FA)+DCI | Placebo/FA | 2.38 [-2.81, 7.57] | 0.37 | / | / | Low |
| FI (pmol/L) | 2 | 1.MI/MI+FA 2.(MI/MI+FA)+DCI |  | -22.20 [-30.58, -13.82] | ＜0.00001 | 0 | 0.64 | Moderate |
|  | 1 | 1.MI/MI+FA 2.(MI/MI+FA)+DCI | Placebo/FA | -23.82 [-34.56, -13.08] | ＜0.0001 | / | / | Moderate |
|  | 1 | 1.MI/MI+FA 2.(MI/MI+FA)+DCI | OCP | -19.68 [-33.07, -6.29] | 0.004 | / | / | Moderate |
| HOMA-IR | 2 | 1.MI/MI+FA 2.(MI/MI+FA)+DCI |  | -0.89 [-1.42, -0.35] | 0.001 | 67 | 0.08 | Low |
|  | 1 | 1.MI/MI+FA 2.(MI/MI+FA)+DCI | Placebo/FA | -1.11 [-1.39, -0.83] | ＜0.00001 | / | / | Moderate |

*Continued*

| **Outcomes stratified by differentcovariates** | **No.  studies** | **Intervention** | **Control** | **Pooled effect size(WD/RR)**  **(95% CI)** | ***P*-value** | **I^2^**  **(%)** | ***P*-heterogeneity** | **Evidencecertainty (GRADE^1^)** |
| --- | --- | --- | --- | --- | --- | --- | --- | --- |
|  | 1 | 1.MI/MI+FA 2.(MI/MI+FA)+DCI | OCP | -0.55 [-1.11, 0.01] | 0.06 | / | / | Low |
| BMI(kg/m²) | 1 | 1.MI/MI+FA 2.(MI/MI+FA)+DCI | Placebo/FA | -0.23 [-1.10, 0.64] | 0.6 | / | / | Low |
|  |  |  |  |  |  |  |  |  |
|  |  |  |  |  |  |  |  |  |
|  |  |  |  |  |  |  |  |  |
|  |  |  |  |  |  |  |  |  |
|  |  |  |  |  |  |  |  |  |
|  |  |  |  |  |  |  |  |  |

*Continued*

| **Outcomes stratified by differentcovariates** | **No.  studies** | **Intervention** | **Control** | **Pooled effect size(WD/RR)**  **(95% CI)** | ***P*-value** | **I^2^**  **(%)** | ***P*-heterogeneity** | **Evidencecertainty (GRADE^1^)** |
| --- | --- | --- | --- | --- | --- | --- | --- | --- |
| 1. MI/MI+FA 2. DCI   3.(MI/MI+FA)+DCI | | | | | | | | |
| TT(nmol/L) | 1 | 1. MI/MI+FA 2. DCI 3. (MI/MI+FA)+DCI | placebo/FA | -0.71  [-1.39,-0.02] | 0.0429 | / | / | Low |
| FT(nmol/L) | 1 | 1. MI/MI+FA 2. DCI   3.(MI/MI+FA)+DCI | placebo/FA | -0.0142[-0.024,-0.0045] | 0.0042 | / | / | Low |
|  |  |  |  |  |  |  |  |  |

| **Outcomes stratified by differentcovariates** | **No.  studies** | **Intervention** | **Control** | **Pooled effect size(WD/RR)**  **(95% CI)** | ***P*-value** | **I^2^**  **(%)** | ***P*-heterogeneity** | **Evidencecertainty (GRADE^1^)** |
| --- | --- | --- | --- | --- | --- | --- | --- | --- |
| Androstenidione(nmol/L) | 1 | 1. MI/MI+FA 2. DCI   3.(MI/MI+FA)+DCI | placebo/FA | -2.4088 [-4.0496,-0.7680] | 0.0039 | / | / | Low |
| SHBG(nmol/L) | 1 | 1. MI/MI+FA 2. DCI   3.(MI/MI+FA)+DCI | placebo/FA | 32.06 [1.27, 62.85] | 0.0413 | / | / | Low |
| DHEAS(µmol/L) | 1 | 1. MI/MI+FA 2. DCI   3.(MI/MI+FA)+DCI | placebo/FA | -2.5115 [-5.5993,0.5760] | 0.11 | / | / | Very low |
|  |  |  |  |  |  |  |  |  |

*Continued*

| **Outcomes stratified by differentcovariates** | **No.  studies** | **Intervention** | **Control** | **Pooled effect size(WD/RR)**  **(95% CI)** | ***P*-value** | **I^2^**  **(%)** | ***P*-heterogeneity** | **Evidencecertainty (GRADE^1^)** |
| --- | --- | --- | --- | --- | --- | --- | --- | --- |
| HOMA-IR | 1 | 1. MI/MI+FA 2. DCI   3.(MI/MI+FA)+DCI | placebo/FA | -0.23 [-1.19 , 0.72] | 0.63 | / | / | Very low |
| AUC Glucose (mmol・min/L) | 1 | 1. MI/MI+FA 2. DCI   3.(MI/MI+FA)+DCI | placebo/FA | -42.3632 [-106.8403,22.1140] | 0.19 | / | / | Very low |
| AUC insulin (pmol・min/L) | 1 | 1. MI/MI+FA 2. DCI   3.(MI/MI+FA)+DCI | placebo/FA | -14452.8923[-19066.2474,-9840.5371] | ＜0.0001 | / | / | Low |
|  |  |  |  |  |  |  |  |  |

*Continued*

| **Outcomes stratified by differentcovariates** | **No.  studies** | **Intervention** | **Control** | **Pooled effect size(WD/RR)**  **(95% CI)** | ***P*-value** | **I^2^**  **(%)** | ***P*-heterogeneity** | **Evidencecertainty (GRADE^1^)** |
| --- | --- | --- | --- | --- | --- | --- | --- | --- |
| BMI(kg/m²) | 1 | 1. MI/MI+FA 2. DCI   3.(MI/MI+FA)+DCI | placebo/FA | -0.45 [-0.89，-0.02] | 0.04 | / | / | Low |
| Pregnancy rates/Clinical pregnancy | 1 | 1. MI/MI+FA 2. DCI   3.(MI/MI+FA)+DCI | placebo/FA | 1.24 [0.85,1.81] | 0.26 | / | / | Low |
